# Gene Model Annotations for *Drosophila melanogaster*: Impact of High-Throughput Data

**DOI:** 10.1534/g3.115.018929

**Published:** 2015-06-24

**Authors:** Beverley B. Matthews, Gilberto dos Santos, Madeline A. Crosby, David B. Emmert, Susan E. St. Pierre, L. Sian Gramates, Pinglei Zhou, Andrew J. Schroeder, Kathleen Falls, Victor Strelets, Susan M. Russo, William M. Gelbart, the FlyBase Consortium

**Affiliations:** * Department of Molecular and Cellular Biology, Harvard University, Cambridge, Massachusetts 02138; † Department of Genetics, University of Cambridge, Cambridge CB2 3EH, United Kingdom; ‡ Department of Biology, Indiana University, Bloomington, Indiana 47405; § Department of Biology, University of New Mexico, Albuquerque, New Mexico 87131

**Keywords:** transcriptome, alternative splice, lncRNA, transcription start site, exon junction

## Abstract

We report the current status of the FlyBase annotated gene set for *Drosophila melanogaster* and highlight improvements based on high-throughput data. The FlyBase annotated gene set consists entirely of manually annotated gene models, with the exception of some classes of small non-coding RNAs. All gene models have been reviewed using evidence from high-throughput datasets, primarily from the modENCODE project. These datasets include RNA-Seq coverage data, RNA-Seq junction data, transcription start site profiles, and translation stop-codon read-through predictions. New annotation guidelines were developed to take into account the use of the high-throughput data. We describe how this flood of new data was incorporated into thousands of new and revised annotations. FlyBase has adopted a philosophy of excluding low-confidence and low-frequency data from gene model annotations; we also do not attempt to represent all possible permutations for complex and modularly organized genes. This has allowed us to produce a high-confidence, manageable gene annotation dataset that is available at FlyBase (http://flybase.org). Interesting aspects of new annotations include new genes (coding, non-coding, and antisense), many genes with alternative transcripts with very long 3′ UTRs (up to 15–18 kb), and a stunning mismatch in the number of male-specific genes (approximately 13% of all annotated gene models) *vs.* female-specific genes (less than 1%). The number of identified pseudogenes and mutations in the sequenced strain also increased significantly. We discuss remaining challenges, for instance, identification of functional small polypeptides and detection of alternative translation starts.

The *Drosophila melanogaster* genome was one of the first metazoan genomes to be sequenced and analyzed (Myers *et al.* 2000) and its manually annotated gene model set has been one of the most carefully curated and highest quality sets available (Misra *et al.* 2002). The updated FlyBase gene model annotations (dos Santos *et al.* 2015) are exported to GenBank annually and serve as the source of the *D. melanogaster* annotations in the NCBI Reference Sequence (RefSeq) database (Pruitt *et al.* 2005). They have also played an important role in informing the gene annotations of subsequently sequenced arthropod genomes by providing a reference set for use with gene prediction algorithms that incorporate a homology-based component.

The strategy behind the FlyBase annotation effort has evolved over time. The initial “stem-to-stern” gene model annotation pass through the entire euchromatic portion of the *D. melanogaster* genome was completed in 2002 (Misra *et al.* 2002) and was based primarily on cDNAs, ESTs, protein homology, and gene prediction algorithms. The next incremental phase of FlyBase annotation involved updating specific gene models in response to new data including cDNAs released by the BDGP (Stapleton *et al.* 2002), community cDNAs, and gene model updates from individual publications. The next phase involved evaluating data analysis triggers. In 2007, numerous reannotation triggers were generated from a comparative genomic analysis of the genomes of 12 *Drosophila* species and phyloCSF, an algorithm designed to identify evolutionary signatures specific to protein-coding genes (Lin *et al.* 2007; Lin *et al.* 2011). This analysis applied to the *D. melanogaster* genome and existing annotation set resulted in the identification of new genes and new isoforms of existing genes, corrections to existing gene models, and the removal of existing coding genes that did not meet the minimal criteria for conservation. Additionally, translation stop-codon readthrough predictions based on the conservation of protein-coding signatures were incorporated (Jungreis *et al.* 2011).

In 2010, RNA-Seq coverage data were assessed as part of our genome annotation effort for the first time using the data from the Baylor College of Medicine Human Genome Sequencing Center (BCM) (Daines *et al.* 2011). In 2011, vast quantities of data on a genome-wide scale from the modENCODE (model organism Encyclopedia of DNA Elements) project (Celniker *et al.* 2009) began to be incorporated into FlyBase. Of the many diverse datasets generated by the modENCODE project (modENCODE Consortium 2010), three in particular were directly incorporated into the FlyBase genome annotation pipeline: the RNA-Seq coverage data (both stranded and unstranded data); RNA-Seq exon junction data; and transcription start site (TSS) profiles (Graveley *et al.* 2011; Hoskins *et al.* 2011). Several other classes of modENCODE data (chromatin domains, transcription factor binding sites, insulators, and RNA-editing sites), have been incorporated into FlyBase and are shown on GBrowse, but they do not directly affect transcript models and have not been assessed relative to the gene annotations.

Given the quantity and quality of the data, and the likelihood that the new transcriptomics data would impact most if not all current annotations, a second complete pass through the entire genome was undertaken to update all of the gene model annotations to the same standard in the context of the new data. This article reflects the annotation set as of the FB2014_06, Dmel annotation release 6.03 (R6.03).

## Materials and Methods

### Philosophy of annotation

For *D. melanogaster*, the body of data informing gene model annotations is extensive and varied, and includes both high-throughput data and detailed characterizations of individual genes. Our goal is to integrate as much of this vast array of data as possible to create a synthesized and consistent set of gene model annotations. (As used by FlyBase, a “gene model” consists of the transcripts and polypeptides produced by a gene; it does not include regulatory elements.) We have reviewed and incorporated millions of pieces of data of widely varying types and quality into our annotations. Evidence types include cDNA, RNA-Seq exon junction, RNA-Seq expression, protein similarity, TSS, and gene prediction in approximately decreasing order of confidence for creating gene models. All data types have strengths and weaknesses and all datasets are prone to artifacts specific to the dataset.

We have taken into account the limitations of the data when creating annotations. Cutoffs and criteria need to be determined for each type of data as curators evaluate the new data types in the context of previously existing data and annotations. Lower-confidence, lower-frequency data may be valuable because it sometimes points to interesting but infrequent variations in gene structure. The difficulty often comes when low-frequency data predict genes structures that do not conform to our expectations for well-behaved genes. Common examples are retained introns and alternative exon boundaries causing frame-shifts, which generally lead to early translation termination and in some cases very short predicted polypeptides. In some cases alternative splicing leads to downstream translation initiation, some of which may actually be unrecognized non-ATG start codons. It is often difficult to determine if these represent artifacts in the data or interesting biology. We have chosen not to represent many of these questionable predicted gene structures as annotations. We require a higher standard of evidence, more than one type of corroborating evidence and/or higher frequency scores, for creating unusual annotations. As more evidence for a previously unusual gene structure (*e.g.*, long non-coding RNAs, noncanonical splices) becomes available, patterns and rules start to take shape, and annotations of these types become more prevalent.

The approach FlyBase has taken differs in some significant aspects from that of the Havana Annotation group, which provides expert manual annotation for human and other vertebrate genomes (Harrow *et al.* 2014). The Havana annotations include detailed documentation of the data that support each component of a main reference variant for each gene, as well as for each variable component for alternative transcripts of the gene. In addition, annotated transcripts corresponding to partial or aberrant cDNAs are included in the gene model, with tags that identify them as problematic. At FlyBase, from the start of the manual annotation effort, the approach has been to create and update gene models to represent the best synthesis of the available data, but not to record the individual pieces of data that contributed to the gene structure (Misra *et al.* 2002). Our philosophy has been that the evidence speaks for itself and the supporting data are easily viewed in GBrowse on the FlyBase web site (http://flybase.org/cgi-bin/gbrowse2/dmel/). As described above, supporting data are vetted; problematic or low-confidence data (including some atypical cDNAs) are not incorporated into the gene model. We have chosen to present a high confidence annotation set and leave the lower confidence variations until such time as there is sufficient evidence to include these alternatives. However, we do not ignore the exceptional (and often inconvenient) cases that are well supported, including polycistronic transcripts, noncanonical splices, and *trans*-spliced transcripts (see Crosby *et al.* 2015, which is the companion to this article). We have chosen not to represent minor variations in gene structure, particularly with respect to 5′ UTR variations. For extremely complex genes capable of producing dozens, hundreds, or, in some cases, thousands of alternative transcripts, we do not represent all possible exon combinations (see *All possible permutations are not annotated*). Our goal has been to produce a biologically accurate, integrated, and maximally useful and usable gene model annotation set.

### Annotation guidelines

To ensure that the gene model annotation is performed as consistently as possible, rules to help curators evaluate the existing evidence were developed during the initial genome annotation effort (Misra *et al.* 2002) and have since been updated periodically (FlyBase Gene Model Annotation Guidelines, http://flybase.org/wiki/FlyBase:Gene_Model_Annotation_Guidelines). The recent influx of high-throughput evidence included novel data types that had not previously been incorporated. Learning to use these new data types for informing gene models was a gradual process, including learning to recognize possible artifacts and reaching agreement among the annotators on how to use the data consistently. In March 2012, a comprehensive revision of our annotation guidelines was completed to accommodate the wealth of high-throughput transcriptomics data and to facilitate consistent application of these updated guidelines to gene model updates and changes.

Explanatory comments have been more consistently applied across the entire annotation set and have been adapted to reflect the incorporation of the new data types (Supporting Information, Table S1). These appear in FlyBase with the gene model on the gene report page and are used to flag unusual aspects of a gene model (unconventional translation start postulated, for example) or as a means of noting data that have not been incorporated into the model. Examples of the latter include “low-frequency RNA-Seq exon junction(s) not annotated” and “annotated transcripts do not represent all possible combinations of alternative exons and/or alternative promoters.” We also use comments at the transcript level, for example, noting if the termini of a transcript are based on RNA-Seq data or, in the case of the 3′ terminus, a polyadenylated cDNA. All gene models have been assessed since R5.45 (FB_2012_03) and have been annotated to these new standards. Our guidelines are still evolving in some areas such as the criteria for creating non-coding RNAs, assessing the protein-coding potential of new transcripts, and using non-ATG translation starts, because general knowledge in these areas is still rudimentary.

As we discuss the various types of high-throughput data and how we used them for annotation, we indicate the associated annotation guidelines that were developed. The principle behind our annotation guidelines is to allow an annotator to create a gene model that best represents the most commonly expressed transcripts of a given gene. In some cases, gene models can be quite complex and may have very many transcripts. In those cases, we often do not annotate every transcript and, instead, we add a gene model comment to indicate that the model is more complex than represented. Data and gene models are available at FlyBase (http://flybase.org).

## Results and Discussion

### Major changes to the annotation set

#### Overview:

Table 1 shows the change in total numbers of various classes of annotations between FB2010_01, R5.24 (the last release before high-throughput data input) and the final release of 2014, FB2014_06, R6.03. Within that time span a new reference genome assembly for *D. melanogaster* became available (Hoskins *et al.* 2015), necessitating the migration of FlyBase gene models from R5.57 to R6.01 (dos Santos *et al.* 2015). Selected statistics relative to each genome annotation release are provided in FlyBase as “Release Notes”; those for prior releases are available in the “Previous Release Notes” section found in “Other Archives” under the Archive tab at FlyBase.

**Table 1:** Annotation statistics for R5.24 and R6.03 FlyBase gene model annotation sets

Feature	Count R5.24	Count R6.03
Genes	14,898	17,684
Protein-coding genes	13,808	13,918
Protein-coding transcripts	21,788	30,385
Exons (protein-coding)	69,091	77,654
Introns (protein-coding)	51,801	58,518
Unique polypeptides	18,295	21,957
Long non-coding RNA genes	133	2446
Long non-coding RNA	158	2871
Pseudogenes	95	301
miRNA genes	90	238
miRNA	90	304

**Figure 1:**
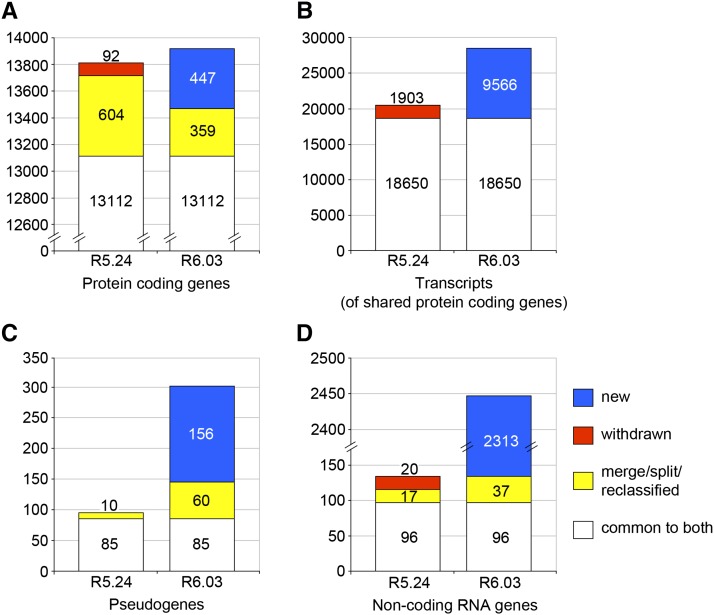
Changes to the FlyBase gene model annotation set in the era of high-throughput data. The gene model annotation set of FlyBase version FB2010_01 (R5.24), the last version to predate FlyBase incorporation of high-throughput data, was compared to that of FlyBase version FB2014_06 (R6.03) to determine the degree of change over the course of 26 annotation updates. Gene model annotations common to both sets (white) were identified. Gene model annotations specific to R6.03 were then examined to identify those derived from R5.24-specific gene model annotations through gene merge/split or reclassification (yellow). The remaining R5.24-specific annotations were classified as “withdrawn” (red), and the R6.03-specific annotations were classified as “new” (blue). The number of gene models within each category of status change is shown for protein-coding genes (A). For 13,003 of the 13,112 protein-coding genes common to both R5.24 and R6.03 (excluding nine complex cases), we also examined the degree to which the number of associated transcripts changed: a measure of gene model complexity. For these genes, the numbers of associated transcripts that persisted (white), were deleted (red), or were added (blue) at some point between R5.24 and R6.03 are shown (B). Changes to the set of annotated pseudogenes (C) and non-coding RNA genes (D) are also shown.

From R5.24 to R6.03, the total number of protein-coding genes increased from 13,808 to 13,918, an increase of only 110 genes (∼0.8%). This modest net change masks a higher degree of flux in protein-coding gene annotations that becomes apparent when individual annotations are tracked between the two releases (Figure 1A, File S1). In this interval, 92 protein-coding genes were deleted, 447 were created, and 604 were split, merged, or reclassified to yield 359 new coding genes. Protein-coding gene annotations that persisted from R5.24 to R6.03 also experienced a large degree of flux. These shared gene models are identified by stable annotation IDs (Table S2), but the number of associated transcripts can change substantially. For the 13,112 genes that persisted between R5.24 and R6.03, 1903 transcript isoforms were deleted and 9566 new transcript isoforms were added (Figure 1B, File S2); new transcripts added to existing protein-coding gene models account for most of the 39% increase in the number of protein-coding transcripts. These changes are largely due to the flood of exon junction data, which led to a significant increase in the number of alternatively spliced transcripts per gene, despite our conservative approach to creating new transcript isoforms. This effect on the predicted proteome is also reflected in the number of unique predicted polypeptides, which increased by 20% from R5.24 to R6.03 (Table 1). The availability of high-throughput data, particularly the RNA-Seq coverage data and the TSS data, also made possible the annotation of UTRs in cases where sparse or no cDNA/EST support had previously precluded any such annotation. As such, the number of mRNA annotations lacking 5′ and/or 3′ UTRs is dramatically reduced (five-fold) in R6.03 compared to R5.24 (Table S3, File S2).

While long non-coding RNA (lncRNA) gene annotations were almost nonexistent in R5.24, there are more than 2400 in R6.03 (Figure 1D, File S1). The number of pseudogenes has tripled (Figure 1C, File S1). It is clear from these broad comparisons that between R5.24 and R6.03, the annotation set underwent major revision; details of the various categories of updates are described in the following sections.

FlyBase relies on outside expert annotations for the various small non-protein-coding classes (miRNA, rRNA, snRNA, snoRNA, and tRNA) as well as natural transposons. Of these, RNA-Seq data led to a great increase in the number of miRNA genes and a more modest increase in the number of snoRNA genes (Berezikov *et al.* 2011, Graveley *et al.* 2011) (Table 1, File S1). The miRNA annotations are imported from miRBase (Kozomara and Griffiths-Jones 2011).

#### Merges and splits:

Gene model merges were the most common category of dramatic change affecting existing gene models based on the new high-throughput data. There have been 211 gene model merges since R5.24, combining 454 different R5.24 annotations into 211 new R6.03 gene models (File S1). Most merges were based on RNA-Seq data, usually RNA-Seq junction data. Such merges often pulled in an upstream 5′ exon to define a new 5′ terminus of the gene (for example, *5-HT2B*) or to create an alternative transcript or transcripts [for example, *Pif1A (Pif1A-RG* and *Pif1A-RH)*, *mwh (mwh-RB* and *mwh-RC)*, *cnc (cnc-RI*, *cnc-RM*, and *cnc-RN)*, *pyd (pyd-RO)*]; adjacent or embedded genes that turned out to be alternative exons within a complex gene model [for example, alternative transcripts of *scrib (scrib-RN* and *scrib-RT)*, *aPKC (aPKC-RG* and *aPKC-RJ)*, *milt (milt-RD* and *milt-RF)*, and *BtbVII (BtbVII-RF)*] were also common. Because junctions spanning related tandem genes are one of the most common types of artifactual junction calls, junctions supporting gene merges can be obscured by artifactual noise. Although the BCM RNA-Seq project (Daines *et al.* 2011) included junctions that could support a merge, the modENCODE project excluded most of them from their original published dataset (Graveley *et al.* 2011). At the request of FlyBase, modENCODE provided a list of all excluded low-confidence junctions; the 1213 junctions that either correspond to a BCM junction (n = 781) or subsequently came to be represented in a gene model annotation because they were supported by other data (n = 432) are included in the FlyBase dataset modENCODE_mRNA-Seq_EXTRA_junctions and are visible on GBrowse.

**Figure 2:**
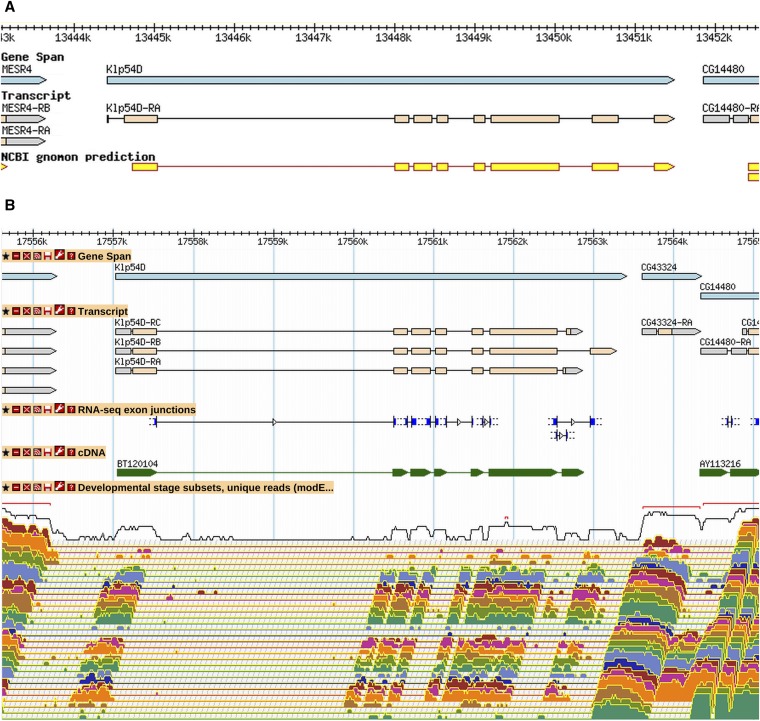
The *Klp54D* gene model was split into two genes. A GBrowse1 view of the *Klp54D* gene model as it existed in R5.30 (A). The gene (blue) and transcript (orange) annotations were based primarily on gene prediction (yellow). On the basis of high-throughput data, this gene model was split in R5.36 to give *Klp54D* and *CG43324*, as shown in an updated GBrowse2 view of this same region, as it exists in R6.03 (B). Below the transcript annotations, modENCODE RNA-Seq exon junctions (blue), aligned cDNA evidence (green), and modENCODE RNA-Seq coverage data for 30 developmental stages spanning early embryogenesis to adulthood are shown from top to bottom. The RNA-Seq expression data show that *CG43324* is expressed at a much higher level and in more stages than *Klp54D*. There is also no RNA-Seq exon junction connecting the two genes. In addition, the annotated 5′ end of *CG43324* is supported by RAMPAGE TSS data (not shown). More information on data presented in GBrowse may be found at http://flybase.org/wiki/FlyBase:GBrowse_Tracks.

Less common than merges were gene model splits: 63 splits that produced 131 new gene models have occurred since R5.24 (File S1). An example of a gene model split based on RNA-Seq data is shown in Figure 2. The original 3′ coding exon of *Klp54D* (based on prediction data) is now annotated as a separate gene, *CG43324*. The RNA-Seq developmental profile indicates that this gene is expressed at additional stages and at higher levels than *Klp54D*. The *CG43324* gene model annotation is also supported by RAMPAGE transcriptional start site data (Batut *et al.* 2013). Note that the newly annotated transcripts of *Klp54D* are also informed by high-throughput data, with alternative 3′ exons supported by RNA-Seq junction data.

#### Alternative transcripts:

Adjusted 5′ start sites, extended 3′ UTRs supported by RNA-Seq coverage data, and additional transcript isoforms supported by junction data are the most common changes to existing gene models. Previously, alternative transcripts were based primarily on cDNA/EST data and published community data. RNA-Seq exon junction data from the modENCODE project and from the BCM group provided a large source of new potential intron/exon boundaries and thus of new transcript isoforms. Of the 71,082 unique exon junctions initially reported to FlyBase, 48,551 were already represented at least once by an intron in existing gene model annotations, leaving 22,531 to be analyzed. After review of these junctions in the context of all available data, another 8841 were incorporated into new transcripts (254 previously incorporated exon junctions were withdrawn). An additional 423 low-confidence modENCODE “EXTRA” junctions are present in R6.03 gene models (File S3).

Of these 9264 newly incorporated junctions, 4172 were exclusively within coding sequences and thus led to the creation of new unique polypeptides, whereas 4861 were exclusively in non-coding RNA or UTR sequences and therefore did not affect coding sequences (Table S4). The majority of exon junctions overlapping UTR or non-coding sequences (69%) were exclusively within 5′ UTRs and many of these were alternative splice donors for existing 5′ exons. In addition, a substantial number of novel 5′ leading exons and, thus, new promoters were annotated on the basis of the RNA-Seq junction data.

For a variety of reasons, 13,953 junctions (12,824 of which overlap 4581 genes) were not included in gene models. Some were clearly artifactual: they spanned tandem repeated genes or were in repeat regions within genes. Many had noncanonical splices that could not be verified (Crosby *et al.* 2015). Some junctions were rejected because they were present at low frequency in very highly expressed genes. Others were not used in annotations because they were low frequency and did not change the gene model significantly or because they created frameshifts and lacked corroborating evidence (see below). The junctions that were not included in a gene model are indicated as “Reviewed, but not incorporated into a FlyBase gene model” in the “Comments” field on the associated junction sequence feature report.

#### Transcription initiation sites:

The annotation of the termini of transcripts is an example of nuanced biological reality that is impractical to capture in detail. Transcription initiation and termination are complex; multiple closely spaced starts and stops often exist for transcripts that are otherwise identical. Based on our annotation guidelines, clustered initiation sites are annotated to a single discrete location and closely spaced polyadenylation sites are annotated as a single 3′ terminus. Thus, a single transcription start site (TSS) and a single polyadenylation site are annotated for the majority of gene models.

Until the modENCODE and other genome-wide TSS data became available, the 5′ ends of the transcripts were set to the 5′ extent of the 5′-most EST or cDNA, except in rare cases when mapped transcription starts from the literature were used (these are cited in the transcript comments). Recent high-throughput data have provided alternative sources for TSSs (Hoskins *et al.* 2011; Nechaev *et al.* 2010; Batut *et al.* 2013), allowing us to refine the annotated 5′ ends of known transcript isoforms and to identify new transcript isoforms; the number of distinct 5′ ends for transcripts has increased more than 25% from 17,576 in R5.24 to 22,082 in R6.03.

**Figure 3:**
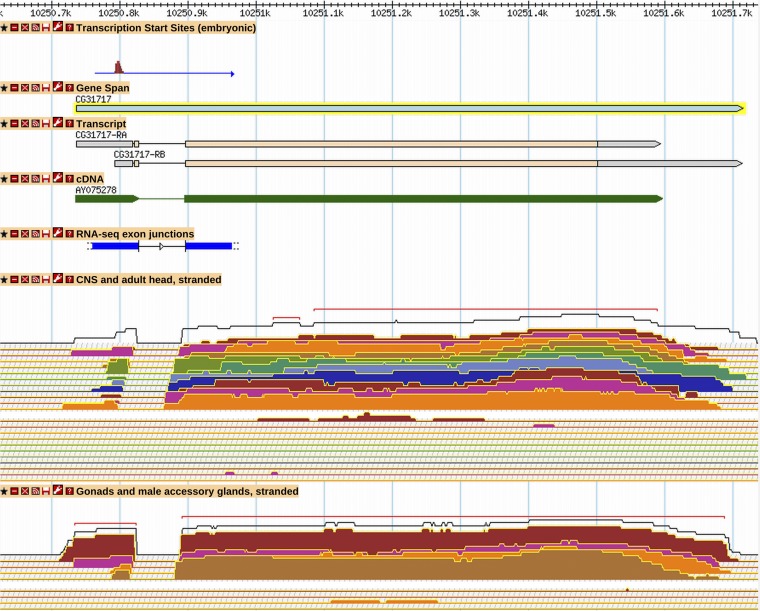
Alternative transcription start site and 3′ end for *CG31717*. A GBrowse2 view of *CG31717*, as it exists in R6.03, depicting (from top to bottom) modENCODE embryonic transcription start site evidence, FlyBase gene and transcript annotations, aligned cDNA evidence, modENCODE RNA-Seq junctions, and modENCODE stranded RNA-Seq expression profiles for CNS tissues (larval, pupal, and adult head samples) and gonadal tissues (testis, accessory gland, virgin female ovary, and mated female ovary); plus strand signal is shown above the minus strand signal for each RNA-Seq track. More information on data presented in GBrowse may be found at http://flybase.org/wiki/FlyBase:GBrowse_Tracks.

FlyBase has systematically reviewed and incorporated into its gene models the modENCODE embryonic TSS data, which mapped TSS regions for 12,454 promoters of 8037 genes (Hoskins *et al.* 2011). These TSS regions were reported as discrete regions containing distributions of transcription starts based on the results of three different mapping approaches. The authors identified 8678 regions supported by at least two of the three types of evidence, which were denoted as “validated,” and FlyBase efforts focused on these sites. The distribution of transcription starts within these TSS regions is complex and, in some cases, the 5′-most mapped start site is actually a nonrepresentative outlier. To define a single, discrete 5′ end for annotated transcripts overlapping a TSS region, FlyBase calculated the “90% TSS point” in each TSS region. This is defined as the point at which 90% of the transcription start signal within the TSS region is encompassed (summation algorithm hits 0.9, starting from the 3′-most TSS and moving 5′). In FlyBase the “Transcription Start Sites (embryonic)” tier in GBrowse displays the full modENCODE TSS region. Between annotation versions R5.24 and R6.03, 222 new TSSs overlapping modENCODE starts were incorporated and 7349 existing start sites were updated to exactly match the 90% point (for example, *CG31717*) (Figure 3). In R6.03, the 5′ end of 14,366 transcripts (43% of all transcripts) match the 90% point of a modENCODE TSS region; in R5.24, only 8586 transcripts (39% of all transcripts) had a 5′ end matching the 90% TSS point or falling within a TSS region (File S4, Table S5). TSS regions defined by Hoskins *et al*. 2011 as “supported” or “5′ RACE only” were not usually supported by other data, and thus they were infrequently used for annotation of transcription start sites.

For 2281 transcripts representing 1682 distinct TSS regions, short-capped RNA data (Nechaev *et al.* 2010) or RAMPAGE promoter profiling data (Batut *et al.* 2013) were used to define the TSS where modENCODE data were absent. The RAMPAGE data were particularly useful because they defined transcription starts throughout the entire *Drosophila* life cycle. For these transcripts, comments are appended that indicate the source of the TSS.

In the absence of any experimental TSS-mapping data, the 5′ extent of the 5′-most EST or cDNA was used, or robust RNA-Seq coverage data were used to estimate the TSS. Five hundred twenty-eight gene models (572 transcripts) still lack any evidence to indicate the extent of the 5′ UTR, and thus the transcript model begins at the translation start site.

#### 3′ UTRs:

One of the most dramatic changes to gene models supported by the RNA-Seq coverage data is the length of 3′ UTRs: a number of genes have alternative transcripts with very long 3′ UTRs, up to 18 kb (Table S6, File S5). In addition, more than 2500 genes have more modest 3′ UTR extensions (Figure 3); these additional transcript isoforms comprise one of the major classes of new alternative transcripts resulting from the assessment of high-throughput data.

Before the RNA-Seq coverage data were introduced, polyadenylated cDNAs were used to set the 3′ extent of transcripts for FlyBase gene models. It is still our policy to represent 3′ termini supported by polyadenylated cDNAs; a comment is appended to the corresponding transcript specifying that this is the case. Where multiple distinct 3′ ends are supported by cDNAs (unless they are within 10 bases of each other), additional isoforms are annotated. More than 12,300 genes, including lncRNA genes (see below), have at least one transcript based on a polyadenylated cDNA. In the absence of a cDNA, the 3′ end is approximated from the RNA-Seq coverage data; in the absence of both cDNA and RNA-Seq data, the 3′ terminus is set to the 3′ end of the translation termination codon.

RNA-Seq coverage data often support 3′ UTR sequences that extend well beyond the longest cDNA. In these cases, at least one transcript is annotated to the approximate terminus of the longest UTR supported by that data and an explanatory comment is appended to the transcript. It is generally not possible to determine which specific isoforms are most likely to be transcribed with the extended 3′ UTR. Thus, a single transcript or a subset of transcripts is annotated with the extended 3′ UTR, although extended transcripts of all isoforms may exist *in vivo*. Multiple polyadenylation sites within the extended 3′ UTR may be present (Smibert *et al.* 2012), but it is not practical to include all such variants, so usually only the longest is annotated. In some cases it is difficult to determine if an extended non-coding sequence corresponds to a long 3′ UTR or an independent lncRNA gene. In fact, both may be true (Mercer *et al.* 2011). A small number of such dual-entity regions have been annotated; the lncRNA is flagged with the comment “This lncRNA overlaps and may be derived from the long 3′ UTR of the upstream gene”; examples include *CR44872* and *CR45040*.

Several groups have performed large-scale analyses that address the phenomenon of alternative transcripts with significantly longer 3′ UTRs. Both Hilgers *et al.* (2011), looking at changes during embryogenesis, and Smibert *et al.* (2012), comparing different larval and adult tissues, observed that transcripts with long extended 3′ UTRs are most frequently present in neuronal tissues. Studies of specific genes subject to regulation by miRNAs, such as *Ubx* (Thomsen *et al.* 2010) and *chinmo* (Wu *et al.* 2012), support the existence of additional miRNA targets within their extended 3′ UTR sequences.

#### New genes (mostly non-coding RNAs):

Previous rounds of annotation focused primarily on protein-coding genes. A limited number of lncRNAs had been annotated based on descriptions from the literature or cDNA evidence. The new high-throughput data, primarily stranded RNA-Seq coverage and exon junction data and to a lesser extent TSS data, provided evidence for many new genes; most of these have been annotated as lncRNAs. Because the majority of regions with any significant coding potential had already been identified, it is not surprising that most new genes appear to be non-coding. Yet, some of these may encode small polypeptides that have yet to be identified or characterized. Because of the uncertainty in this area, new genes that are annotated as non-coding are generally commented as “probable lncRNA gene; may encode small polypeptide(s),” whereas some that are designated as coding are given the comment “possible non-coding RNA gene.” In R5.24, only 133 non-coding RNA genes had been annotated. From R5.24 to R6.03, 2313 new candidate non-coding genes were annotated (including those flagged as antisense, see below).

We assessed the proposed lncRNAs described in the published literature (Tupy *et al.* 2005; Inagaki, *et al.* 2005; Hiller *et al.* 2009; Young, *et al.* 2012) and annotated many, but not all, of the lncRNAs proposed. Unless we had independent evidence that the region is transcribed (for example, RNA-Seq coverage data), we did not annotate the predicted lncRNA gene. In some cases, we found that the putative lncRNA corresponded to an extended 3′ UTR of an existing gene; in others, we found that there was evidence of a small functional ORF. In these cases, the symbol or designation used in the publication was captured as a synonym of the corresponding coding gene.

Two systematic genome-wide searches were performed to identify additional regions containing potential new genes or new transcribed regions. First, we analyzed a list of intergenic RNA-Seq junctions, some of which were incorporated into new gene models. More often, intergenic junctions were indicative of new 5′ extents of existing genes. In some cases, it was impossible to incorporate such a junction into a gene model due to the lack of any additional or consistent evidence. Additional evidence might include ESTs, consistent RNA-Seq coverage data, or transcription start site data. Intergenic junctions that were not included in a gene model were marked with a comment.

**Figure 4:**
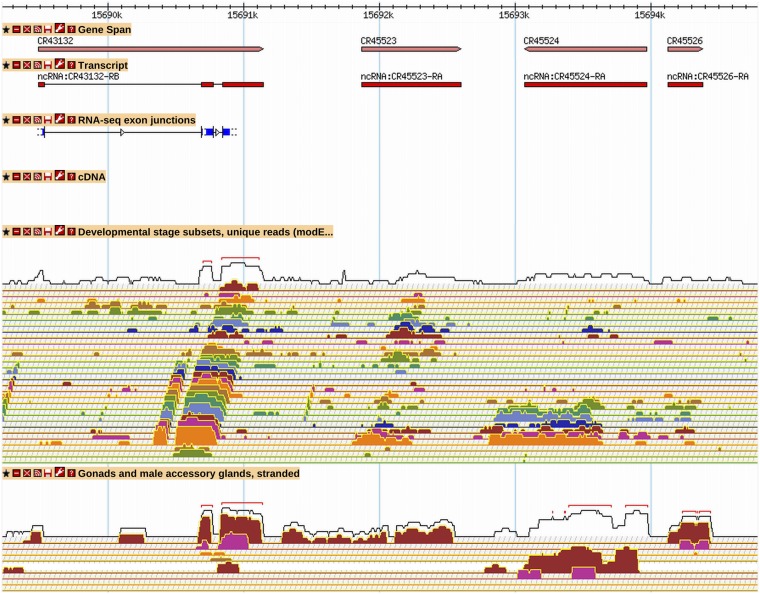
New long non-coding RNA genes are supported by RNA-Seq data. A GBrowse2 view for a region containing four recently annotated lncRNA genes is shown (R6.03). *CR43132* is supported by RNA-Seq junction and expression data. *CR45523*, *CR45524*, and *CR45526* are supported by RNA-Seq expression data only; they were identified in a genome-wide scan for intergenic regions with RPKM values of 3 or more. The transcript polarity is determined from the stranded “Gonads and male accessory glands” RNA-Seq expression tracks. *CR45523*, *CR45524*, and *CG45526* show expression primarily in male testis (red RNA-Seq signal), a pattern common to many of the newly annotated ncRNA genes. See Figure 2 and Figure 3 for GBrowse track descriptions. More information on data presented in GBrowse may be found at http://flybase.org/wiki/FlyBase:GBrowse_Tracks.

A second method to identify new genes was based on analysis of intergenic RNA-Seq coverage values calculated as reads per kilobase per million reads (RPKM) (Mortazavi *et al.* 2008). The genome was scanned step-wise in 200-bp windows for regions with an RPKM value of 3 or more. If the RNA-Seq coverage data (with or without supporting TSS data) made it possible to determine discrete transcript ends and was present in more than one RNA-Seq data track, then a new gene was annotated (Figure 4). Although many new genes were created in this way, we found that establishing absolute criteria for making a new gene was difficult, and that discerning noise from the real RNA-Seq signal was not always possible.

Short predicted ORFs (<50 aa) are extremely frequent in the genome, just by chance, but it is challenging to identify which of these are translated *in vivo*. In the past, we made use of the PhyloCSF metric that aims to identify conserved protein-coding signatures (Lin *et al.* 2007; Lin *et al.* 2011). The sensitivity of this algorithm falls off for very small proteins (roughly less than 35 amino acids); for example, this analysis failed to detect the ORFs of *SclA* and *SclB*, which are highly conserved polypeptides 28 and 29 amino acids in length (Magny *et al.* 2013). The PhyloCSF analysis was also quite conservative, requiring conservation across 11 *Drosophila* species, including several in the *Drosophil*a subgenus. When creating new gene annotations supported by RNA-Seq data, we assessed the longest ORF, requiring conservation only within the melanogaster subgroup (which is in the Sophophora subgenus), including conservation of the initiating methionine and absence of frameshifts or internal stops. These case-by-case informal assessments were neither rigorous nor exhaustive; the smallest polypeptide annotated using this approach was 32 amino acids. Even for transcripts that have been accurately identified as containing a short translated ORF, the picture may be incomplete; many small proteins characterized thus far are encoded on polycistronic transcripts. Without any additional high-throughput predictive tools, we have relied on the published literature to point us to specific cases of very small proteins (Wolfner *et al.* 1997; Savard *et al.* 2006; Kondo *et al.* 2007; Galindo *et al.* 2007; Hayden and Bosco 2008; Findlay *et al.* 2009; modENCODE Consortium 2010; Ladoukakis *et al.* 2011; Magny *et al.* 2013; Brown *et al.* 2014).

Ladoukakis *et al.* (2011) identified 401 small ORFs (<100 aa) that, by their criteria, appeared likely to encode functional polypeptides. However, they limited their assessment of conservation of ORFs to a comparison of *D. melanogaster* and *D. pseudoobscura*. They also failed to distinguish between conservation at the nucleotide *vs.* the protein level. We extended the analysis to additional sequenced species within the Sophophora subgenus (*Drosophila* 12 Genomes Consortium 2007), but we found that this type of analysis is not informative for most ORFs less than 25–30 aa. Of the 50 ORFs we found to show conservation consistent with a protein-coding extent, many correspond to alternative or 5′ exons of larger genes or to cases of stop-codon readthrough (Jungreis *et al.* 2011). Nine appear to encode small polypeptides, including four that had been previously identified. Three of the uniquely identified small protein candidates correspond to small conserved ORFs within the 5′ UTRs of larger genes. This finding underscores one of the difficulties in identifying ORFs for very small proteins: they may share transcripts that have already been annotated with protein-coding extents. One of the newly identified genes, *CG43732* [referred to by Ladoukakis *et al.* (2011) as Dm_3R:78579), appears to be conserved among the Dipterans, including mosquitos, and perhaps among other insects as well (Tribolium, GenBank accession EEZ97819).

Of the new gene models created since R5.24, 63 are annotated as coding genes for which the largest predicted polypeptide encoded is 50 amino acids or fewer. Of these, 51 encode polypeptides between 30 and 50 amino acids and 12 encode polypeptides less than 30 amino acids in length. The total number of genes encoding polypeptides of 50 amino acids or less in R6.03 is 108; 18 of these encode polypeptides of less than 30 amino acids (File S6).

#### Antisense transcripts:

**Figure 5:**
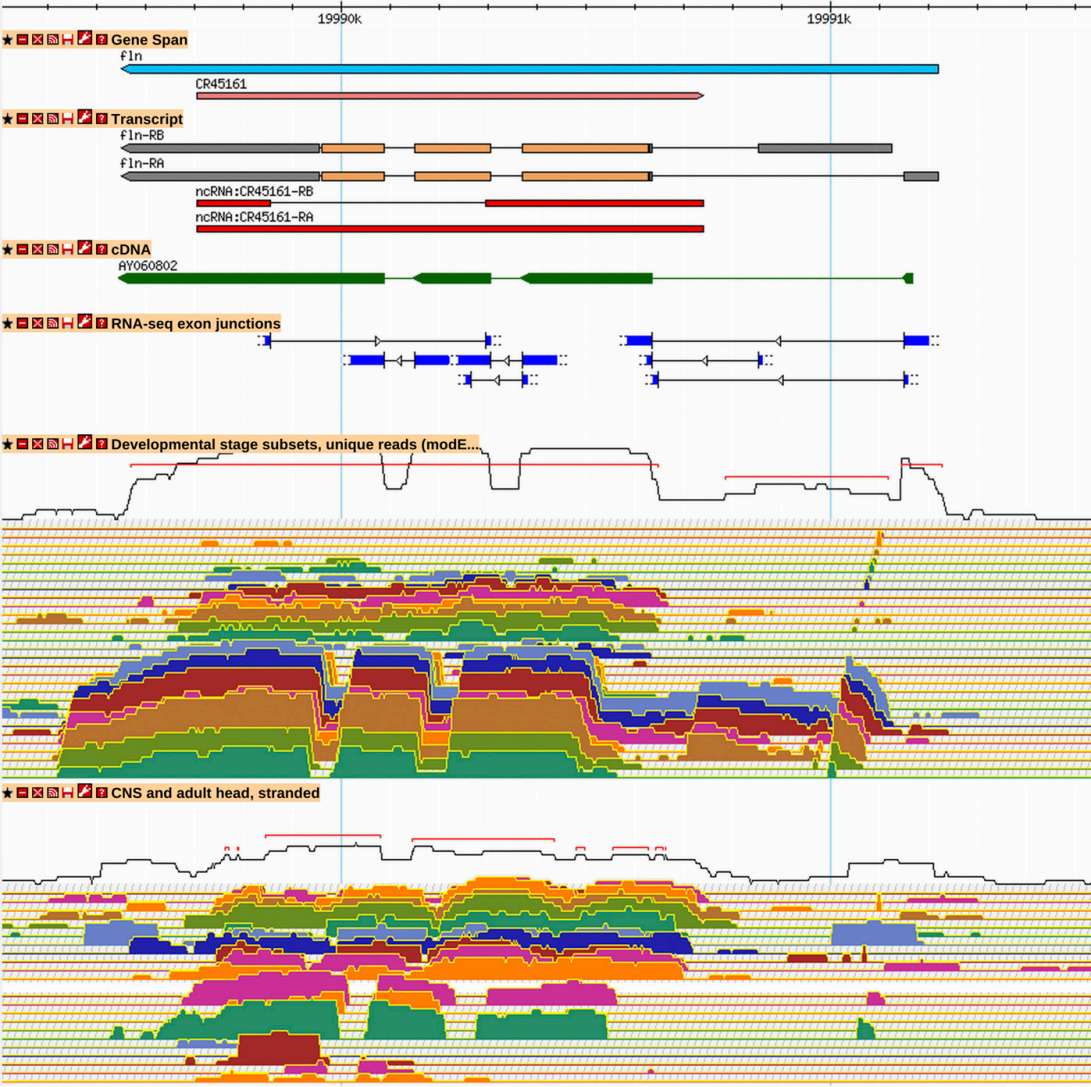
New ncRNA gene *CR45161* is antisense to *fln*. *CR45161* is a newly annotated antisense gene supported by RNA-Seq expression and junction data. Although it might be mistaken for background transcription in the unstranded “Developmental stage” RNA-Seq expression tracks, its strong transcription on the positive strand is obvious in the stranded “CNS and adult head” RNA-Seq track. See Figure 2 and Figure 3 for GBrowse track descriptions. More information on data presented in GBrowse may be found at http://flybase.org/wiki/FlyBase:GBrowse_Tracks.

In the past, an occasional cDNA provided evidence for a lncRNA gene with extensive regions of antisense transcription. When RNA-Seq junction data and stranded RNA-Seq coverage data became available, it became clear that such antisense genes are not rare. In R6.03, there are 476 non-coding RNA annotations annotated with the SO term "antisense_gene" (SO:0000077) (Figure 5). This represents almost 20% of annotated lncRNAs. We have not yet completed a systematic search for antisense transcription based on RPKM values and expect that more antisense genes will be identified.

Only one case of overlapping coding regions on opposite strands has been annotated. Both genes (*CG34148* and *P5CDh2*) encode proteins of known functions that are well conserved outside of *D. melanogaster*. However, 323 loci (667 genes) that share overlapping CDSs on opposite strands were recently reported (Brown *et al.* 2014). Their proposed new protein-coding extents on antisense transcripts were identified on the basis of conservation with other *Drosophila* species, but such conservation may simply be the result of a shadow effect due to shared nucleotide sequence with a highly conserved CDS on the opposite strand. For that reason the newly identified antisense transcripts are annotated as non-coding genes in FlyBase.

#### Sex-specific (or highly sex-biased) gene expression:

During the annotation process, as we examined the RNA-Seq coverage data available for developmental stages and tissues, it became clear that new male-specific genes were relatively common, but new female-specific genes were rare. Using the FlyBase RNA-Seq Search tool (based on binned RPKM values, see description at http://flybase.org/reports/FBrf0221009.html), we were able to quantitate this informal observation. The total number of male-specific genes, ∼2400 including ∼770 lncRNA genes, dramatically exceeds the total number of female-specific genes: ∼130 with very few lncRNA genes (precise numbers depend on the specified criteria; antisense genes are included in the lncRNA counts; File S7). Using broad criteria with no requirement for gonadal expression and inclusion of possible maternal-effect genes to tabulate the number of newly annotated (since R5.24) genes, highly male-biased expression is observed for 171 coding genes and 753 lncRNA genes; highly female-biased expression is observed for 11 coding genes and four lncRNA genes. Assessing all genes showing sex-biased expression, only four male-specific genes show no appreciable expression in testis (*CG6788*, *CR43451*, *CR43942*, *CR45400*). A number of other male-specific genes not expressed in testis appear to exist that have not been annotated due to lack of strand information, but the total is estimated to be less than 1–2% of all male-specific genes. In contrast, 19 female-specific genes (∼15%) show no appreciable expression in ovaries. These include genes expressed in the spermatheca and two genes required for pheromone biosynthesis (*Fad2* and *eloF*). Almost half of the female-specific genes also show high transcript levels in early embryos.

By the criteria used, only five lncRNAs appear to be female-specific; three of these share some interesting characteristics. The most dramatic is that they span extremely long genomic ranges, more than 220 kb in the cases of *CR32773* and *CR44357* and 76 kb in the case of *CR43836*, despite having processed transcripts of modest size (less than 2.5 kb). All three are expressed at low to moderate levels in ovaries and embryos; all are located on the X chromosome and produce multiple transcripts due to variable use of alternative exons. Genes with very low levels of expression are excluded in the analysis of sex-biased expression described above; among these are three additional apparently female-specific lncRNA genes with very long genomic extents, all on the X chromosome (*CR44999*, *CR44894*, and *CR43960*). Non-coding genes that span long genomic extents are relatively uncommon. These six genes comprise the majority of annotated lncRNAs transcribed from genomic regions more than 72 kb in length. There are only four others: *iab-8*, *CR44833*, *CR44997*, and *flamenco*, a piRNA-producing locus.

### Limitations of the data and the approach

#### Not all transcript isoforms with alternative splices within UTRs are annotated:

After review, 13,944 RNA-Seq exon junctions were not incorporated into gene annotations. Many of these are junctions that support alternative splices within 5′ UTRs representing only minor variations of a few bases in a splice donor or acceptor. In light of our goal to produce a useful and usable annotation set, we are reluctant to create gene models with many alternative transcripts that differ by only small changes in the 5′ UTRs. Thus, for a given TSS, only a subset of such splices may be annotated. In other cases, predicted junctions in UTRs are not annotated because they are present at a low frequency relative to other junctions in the gene (based on read-counts) or have low confidence scores (Graveley *et al.* 2011). Whenever junction data were not incorporated in the gene model, comments were added to the gene record to indicate that the annotated transcripts do not represent all supported alternative splices within the 5′ UTR or that additional RNA-Seq low-frequency junctions exist that were evaluated but not annotated.

#### Protein isoforms supported only by low-frequency junction data are omitted:

Many junctions that were not annotated were within coding sequences. The general rule in these cases was to create a new transcript isoform if the new junction is present at a frequency of 1% or more of the highest junction frequency within the gene, unless the new junction leads to a frameshift, in which case the threshold was set to 10%. When merited, a comment indicating that low-frequency junctions were not annotated was appended.

Our conservative criteria for annotating alternatively spliced isoforms based on RNA-Seq junction data have implications for the number of unique 5′ exons, the overall number of transcript and protein isoforms annotated, and the “permutation problem,” as described below.

#### All possible permutations are not annotated:

**Figure 6:**
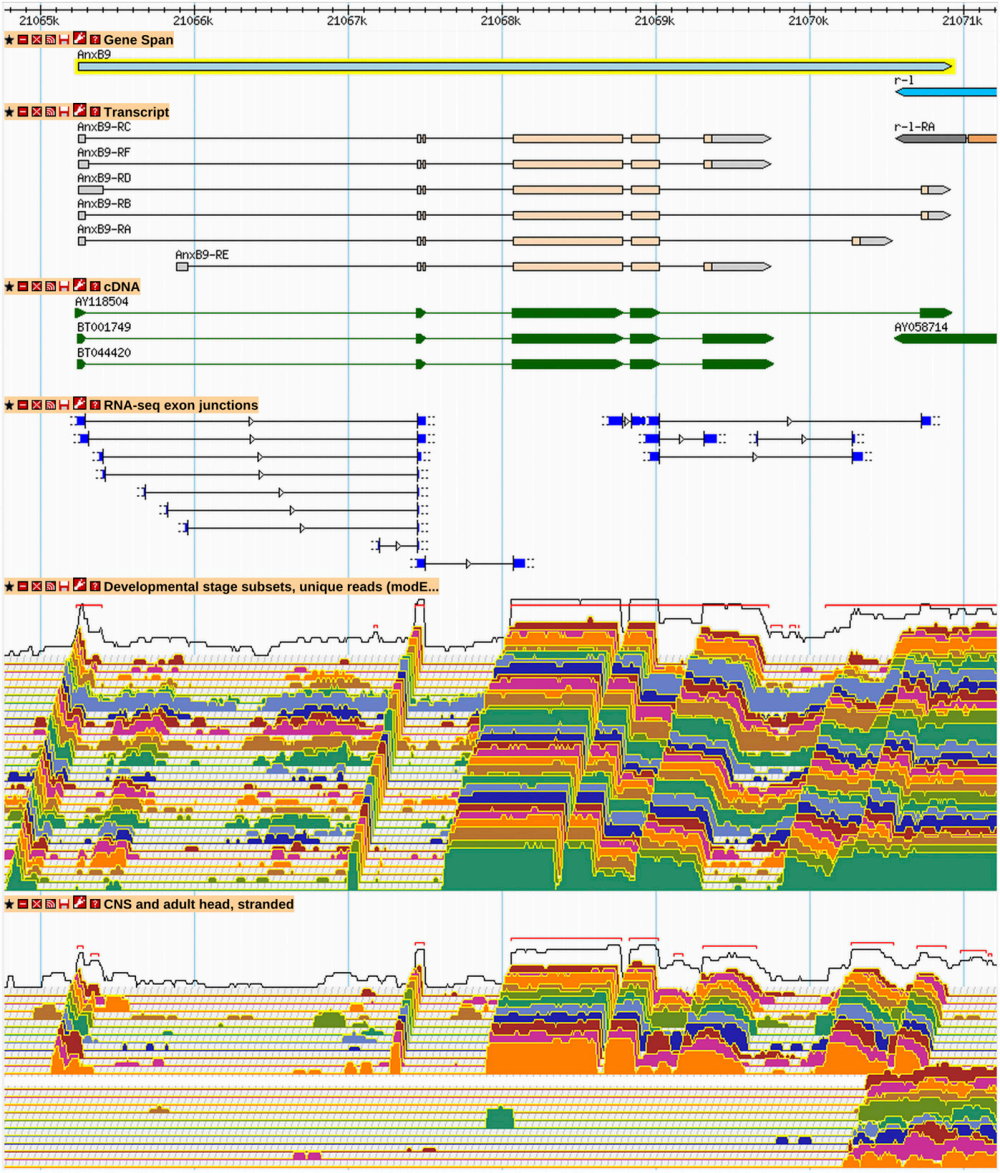
A subset of possible *AnxB9* transcript isoforms has been annotated. RNA-Seq junction and expression data predict eight alternative splice donors from three different leading 5′ exons, of which four have been used in annotations. Low-frequency junctions have not been annotated. Alternative splicing in the last intron leads to three different protein isoforms. A low-frequency junction at the 3′ end of the gene has also been excluded. Twelve different transcript isoforms are possible using the annotated junctions (32 are possible with all junctions), but only a subset of the possible combinations has been annotated. See Figure 2 and Figure 3 for GBrowse track descriptions. More information on data presented in GBrowse may be found at http://flybase.org/wiki/FlyBase:GBrowse_Tracks.

If noncontiguous data, such as RNA-Seq junction, EST, and TSS data, support alternative exons in several regions of a gene, it is usually not possible to determine which of the possible exon combinations actually exist *in vivo* as transcripts. We call this the “permutation problem.” All configurations of exons supported by full-length cDNAs are annotated (unless they involved retained introns). The number of additional transcripts created was at the discretion of the annotator. Excluding low-frequency junctions, all alternative splices within the CDS and all promoters have been represented at least once, but not necessarily all possible combinations of available exons were combined into transcripts. An example of a gene for which all possible annotations supported by junction data have not been annotated is *AnxB9* (Figure 6). Generally, our approach was to annotate using the high-frequency RNA-Seq junctions in the majority of transcripts and to represent the low-frequency junctions in at least one transcript isoform. For cases in which all combinations were not created, a comment to that effect was included in the Gene Model Comment section of the gene report.

The decision not to attempt to create all 5′ exon alternatives and all exon permutations was based on both biological and practical considerations. As stated above, with available data, it is often not possible to determine which exon combinations are transcribed in the organism. In addition, it would be completely impractical to manually annotate large numbers of transcripts using currently available tools or to represent them in the GBrowse user interface. This policy led to a significant under-representation of transcripts in some instances, particularly for the ∼50 genes that have the capacity to encode more than 1000 transcript isoforms each, *e.g.*, *Dscam1*, *cpx*, *para*. For a more comprehensive compilation of predicted transcripts, see Brown *et al.* (2014).

### Challenging annotation types

#### Mutations in the sequenced strain:

The sequenced *D. melanogaster* strain (*iso-1*, Bloomington stock #2057) (Brizuela *et al.* 1994) has a set of previously known visible mutations: *y^1^*, *cn^1^*, *bw^1^*, *and* *sp^1^*. The mutational lesions corresponding to the first three markers were readily identified; the gene model annotation that corresponds to the *sp* (*speck*) locus still has yet to be determined. During the course of annotation, many more genes were found to have mutations relative to other strains of *D. melanogaster* and/or closely related *Drosophila* species. Currently, 55 genes models are annotated with the comment “Mutation in sequenced strain” or “Known mutation in sequenced strain” (File S8); 11 of these may be polymorphic pseudogenes (see below). Of the 55 identified genes, 10 have an inserted transposon, four have a point mutation in the initiation codon or a splice site, 15 are characterized as having nonsense mutations or a premature stop, 12 are frameshifts, 11 are deletions, and three are “complex,” having multiple lesions. Interestingly, 33 of the 55 genes display more than 50% sequence similarity to another gene in the genome, which may explain in part why so many mutations are tolerated in this strain. An additional four gene models are labeled “Possible mutation in sequenced strain”; these correspond to transposon insertions in introns or immediately adjacent to genes. The mandate of the annotation project is to present the wild-type proteome of *D. melanogaster*. To this end, the mutant polypeptide sequences in these cases have been replaced with a “wild-type” sequence in FlyBase. These are indicated with an exception and explanatory note in the GenBank protein records. The reported transcript sequences, however, correspond to the mutant genomic sequences of the sequenced strain (*iso-1*).

Some of the "mutations in strain" might be more accurately described as variants (see discussion of polymorphic pseudogenes, below). A particularly interesting mutational variant present in the sequenced strain is the insertion of a Doc transposable element in the C*HKov1* *g*ene (*CHKov1^Doc1420^*). In wild populations this variant has recently increased in frequency under directional selection (Aminetzach *et al.* 2005). It has been determined that two useful phenotypes segregate with the insertion: increased resistance to organophosphate pesticides (Aminetzach *et al.* 2005) and resistance to sigma viral infection (Magwire *et al.* 2011).

#### Pseudogene annotations:

Pseudogenes are typically defined as regions with similarity to a functional gene but containing lesions that preclude the generation of the functional product encoded by the "parental" gene, usually a protein or a structural RNA. For the identification of pseudogenes related to non-coding structural RNAs, FlyBase relies on external, published analyses. For pseudogenes of protein-coding genes, FlyBase genome annotators examine the predicted effects of lesions on the predicted polypeptide. Pseudogene classification requires evidence of neutral selection; in practice, we take as evidence of neutral selection two compromising lesions (*e.g.*, nonsense or frameshift mutations). Genes with a single disabling mutation in the sequenced strain are annotated as coding genes and marked as "mutant in strain" (see section above). In some cases, a gene may have two disabling mutations in certain strains but appear to be wild-type in others. A good example is *Est-P*, a gene that exhibits strain-specific disruptions to the coding sequence and has a conservation rate lower than the parental *Est-6* gene (Balakirev and Ayala 2004). Such genes are classified as "polymorphic pseudogenes"; they are also annotated as "mutant in strain," with a gene model comment explaining their nature. Most annotated polymorphic pseudogenes in FlyBase have come from analyses of gustatory, olfactory, and ionotropic receptors (Robertson *et al.* 2003; Robertson 2009; Croset *et al.* 2010).

Using these criteria, FlyBase has annotated 301 pseudogenes: 30 are rRNA or tRNA-derived, and the remainder are derived from protein-coding genes. Pseudogenes are marked in FlyBase by the SO term "pseudogene_attribute" (SO:000042). Additionally, a gene model comment describes the parental gene and type of duplication: “Pseudogene similar to [parental gene]; proximate/transposed/retrotransposed.” Compared to R5.24, the 301 pseudogenes represent a tripling, mostly due to the identification of transcribed pseudogenes using transcriptomic data. With the recent release of a new genomic sequence assembly, we are finding additional pseudogenes that are not reflected in these numbers, especially on the newly assembled Y-chromosome. We note that automated annotation algorithms such as GRIT (Brown *et al.* 2014) are not set to differentiate functional genes *vs.* pseudogenes, so manual annotation provides added value in this respect.

It is notable that the number of pseudogenes annotated by FlyBase pales in comparison to the thousands annotated for the human genome by GENCODE (Sisu *et al.* 2014). In addition, while there are thousands of processed (retrotransposed) pseudogenes annotated for the human genome, there are only six annotated in *D. melanogaster (*"processed_pseudogene," SO:0000043). In part, this reflects differences in biology. Retrogene bursts have been characterized for several mammalian lineages (Pan and Zhang 2009), whereas pseudogenes are known to be relatively rare in *Drosophila*, by comparison to other eukaryotes, due to strong selection in intergenic regions and high deletion rates (Harrison *et al.* 2003). The low number of FlyBase annotated pseudogenes may also reflect a conservative annotation approach. Our goal has not been to annotate all possible gene fragments exhaustively, but rather to annotate as pseudogenes those regions that would otherwise be mistaken for functional protein-coding genes.

#### Annotation of alternative transcripts that encode significantly shorter polypeptides:

A surprising number of genes have evidence supporting alternative transcripts that either produce significantly shorter protein isoforms or are non-coding transcript isoforms. These typically have a retained intron, an alternative 3′ exon, an alternative splice that results in a premature stop, or the lack of a 5′ coding exon present in other transcripts, requiring a downstream start. In some cases, there is published evidence that these are functional protein-coding transcripts, including verification of the resulting short protein isoform on Western blots (for example, *dysc*, Jepson *et al.* 2012; *nsl1*, Yu *et al.* 2010; *Adar*, Chen *et al.* 2009; *vari*, Bachmann *et al.* 2008). These cases are flagged in FlyBase with comments that include the phrase “short protein isoform supported” in the transcript reports. Some loci appear to encode both coding and non-coding transcripts. One well-studied case is an RNA-splicing factor for which partially processed transcripts with retained introns are relatively stable; they appear to be retained in the nucleus and thus are not translated [su(w^a^)] (Chou *et al.* 1987; Zachar *et al.* 1994). It is interesting to note that a number of other RNA-splicing factors or spliceosome components produce abundant transcripts with retained introns (for example, *B52*, *CG1646*, *Moca-cyp*) or alternative exons that disrupt the CDS (*Caper*). Another mechanistic possibility is that some cells can tolerate a relatively high level of aberrant transcripts, and some of these transcripts are simply biological noise. Such aberrant transcripts may be targets of nonsense-mediated decay (NMD). However, while the genes effecting NMD are highly conserved and are present in *Drosophila* (Behm-Ansmant, *et al.* 2007), genes identified as direct targets of NMD in *Drosophila* were not found to have a higher than average level of premature termination codons (Chapin *et al.* 2014). For all but a few dozen well-studied genes, which of these several possibilities may be true cannot be determined by assessment of the available data.

For transcripts of this type that encode anomalously short polypeptides, we require a higher standard of support to merit annotation. Transcripts with retained introns may be annotated in cases where there are both supporting cDNA and RNA-Seq coverage data. Many low-frequency, low-confidence score RNA-Seq junctions have been found to throw the CDS out of frame and lead to premature termination codons; we typically do not annotate such junctions. Junctions present at a level of 10% or more relative to the highest junction frequency for any junction within the gene or junctions that are also supported by cDNA data have been annotated, even when they lead to early translation termination. An explanatory comment that includes “may or may not produce functional polypeptide” is included in the transcript report. Even based on these very conservative criteria, more than 700 gene models include transcripts flagged with such a comment. By contrast, the GENCODE consortium, which aims to annotate all gene features in the human genome, includes all transcripts predicted to generate truncated polypeptides. In the GENCODE 7 reference annotation set (Harrow *et al.* 2012), 44.9% of the translated isoforms (59.9% of the alternative isoforms) would lose either functional or structural domains or functional residues relative to the constitutive isoform (as defined in the APPRIS database; http://appris.bioinfo.cnio.es/).

#### Alternative transcripts with nonoverlapping CDSs:

There are a number of gene models with short protein isoforms with a counterintuitive characteristic: pairs of alternative isoforms that encode nonoverlapping protein products. Both members of the pair are considered parts of one gene model because longer isoforms that are well-supported overlap both. In 22 of 30 such cases, these nonoverlapping protein isoforms are encoded by nonoverlapping transcripts (*i.e.*, with their own well supported and distinct 5′ transcription start and 3′ polyadenylation sites). Such gene models have been flagged with the comment, “Gene model includes transcripts encoding nonoverlapping portions of the full CDS.”

**Figure 7:**
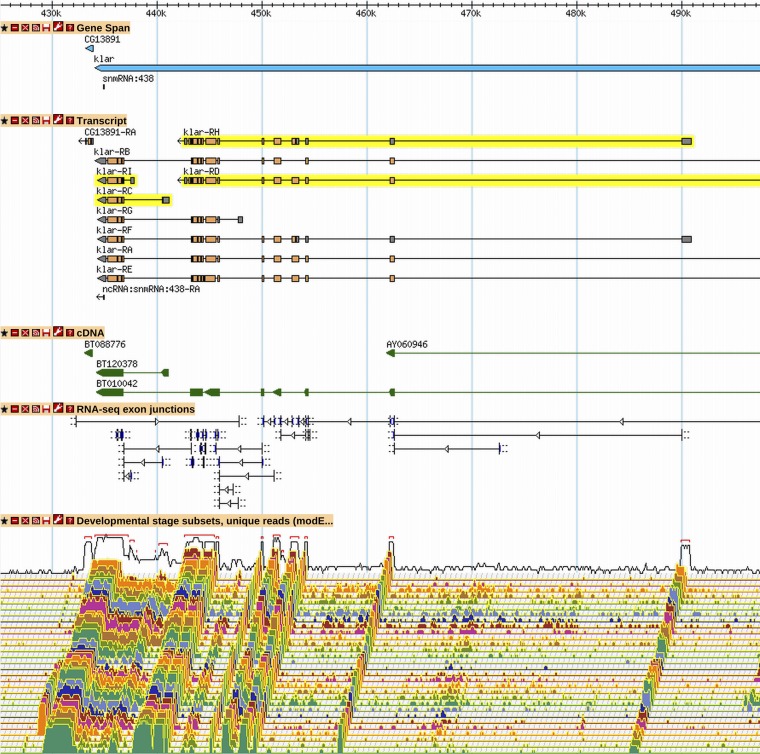
The two nonoverlapping protein isoforms of *klar*. A GBrowse2 view of *klar* is shown, as it exists in R6.03, with nonoverlapping isoforms highlighted in yellow (*klar-RC* and *-RI* do not overlap *klar-RD* and *-RH*). The C-terminus of the longer, "upstream" isoforms (*klar-RD* and *-RH*) is sufficient for targeting proteins to lipid droplets, whereas the "KASH" domain present in the "downstream" isoforms (*klar-RC* and *-RI*) is sufficient for targeting to the nuclear envelope (Guo *et al.* 2005). The "upstream" nonoverlapping isoform is necessary for proper lipid droplet targeting in the embryo. While the KASH domain is necessary for nuclear migration in the embryo and retina, this function is associated with the "full-length" KASH-containing isoforms. The short KASH-containing isoform, which lacks motor interaction domains, is expressed (Western blot, immunofluorescence) and is apparently enriched in nurse cells but is not sufficient to rescue nuclear migration in the retina. See Figure 2 and Figure 3 for GBrowse track descriptions. More information on data presented in GBrowse may be found at http://flybase.org/wiki/FlyBase:GBrowse_Tracks.

Six of these cases have been identified in the literature, but in no case has the functional significance of each of the two nonoverlapping isoforms been fully characterized. The best characterized case is the *klar* gene (Figure 7). The *klar* protein has been implicated in microtubule-based transport of lipid droplets (Patterson *et al.* 2004) and nuclei (Guo *et al.* 2005). The existence of nonoverlapping transcripts encoding nonoverlapping protein isoforms has been characterized by cDNA analysis, RT-PCR, and Western blot (Guo *et al.* 2005; Kim *et al.* 2013).

### The future: more data and new approaches

#### Additional transcriptome data:

Although we have been spoiled with an abundance of transcriptome data, there are some gaps that may be minimized in the future. For example, while stranded RNA-Seq data have been available for larval and adult tissues, the data originally available for the developmental profiles are unstranded. This has compromised our annotation of lncRNAs expressed only in embryonic or early larval stages, which we will be able to correct now that stranded data for these stages are in the pipeline. The reverse problem affects annotation of transcription start sites: modENCODE TSS data are heavily weighted toward embryonic RNA samples. We have made some use of RAMPAGE TSS data (Batut *et al.* 2013), which are drawn from a complete range of developmental stages, and will soon make more systematic use of that dataset. Additional RNA-Seq junction data from defined tissues may reveal tissue-specific splicing that would allow more accurate annotation of alternative transcript isoforms.

#### Small RNAs associated with distinct genomic regions:

Some classes of abundant small RNAs cannot be practically annotated as distinct transcripts or genes; these include piRNAs (reviewed in Senti and Brennecke 2010) and endogenous siRNAs (Okamura *et al.* 2008; reviewed in Piatek and Werner 2014). However, the chromosomal regions from which these two classes of small RNAs are derived can be defined (for example, Brennecke *et al.* 2007; Wen *et al.* 2014; Brown *et al.* 2014). We plan to add annotations of such regions as sequence features that can be viewed in GBrowse.

#### Confirming annotated translation starts and confronting uORFs:

Most of the high-throughput data we have used to inform our gene models define transcripts or transcribed regions, not translated regions. This may be about to change. Techniques being developed in vertebrate systems, notably ribosome profiling (Ingolia *et al.* 2011; Chew *et al.* 2013), may soon be applied to *Drosophila*. Using a modification of ribosome profiling that leads to accumulation of ribosomes at sites of translation initiation, Ingolia *et al.* (2011) detected multiple cases of misidentified translation starts or alternative translation starts. These studies also identified a large number of out-of-frame alternative starts in 5′ UTRs, some of which may correspond to functional upstream ORFs (uORFs). Whether these results represent experimental false positives or biological noise, or are biologically relevant, remains to be determined (Chew *et al.* 2013; Ingolia *et al.* 2014; Pauli *et al.* 2014).

Our initial annotations of translation starts depended on predictions, protein homology, and cDNA data. In the absence of obvious BLAST alignment data to the contrary, our 2007 Gene Model Annotation Guidelines specified that we always set the start codon to the most upstream ATG. This has changed little in the subsequent years. The PhyloCSF exon prediction algorithm allowed us to determine whether annotated ATG sites were conserved and led to the reannotation of 164 transcripts with a downstream translation start site. When a downstream ATG site is annotated, we add a comment to the transcript explaining the annotation. A small number of genes (27) have been annotated with a noncanonical translation start (Crosby *et al.* 2015) and are flagged with comments at the gene and transcript levels. As discussed in Crosby *et al.* (2015), we suspect that there are many more noncanonical translation starts, and possibly numerous undetected alternative translation starts. We have made no attempt to annotate uORFs, except in cases where they show evidence of encoding conserved polypeptides (Lin *et al.* 2007; Hayden and Bosco 2008).

#### Very small polypeptides or long non-coding RNAs:

One of the most difficult decisions we often had to make was whether to annotate a gene supported by high-throughput transcriptome data as coding or non-coding (see section above on annotation of new genes). This question is at the intersection of two aspects of biology about which very little is known: the roles of lncRNAs and of very small proteins. High-throughput studies have utilized various algorithms to detect conservation of short ORFs. These approaches have failed to reliably detect proteins less than 30 amino acids and, of course, proteins that are not conserved will not be detected. Until translation-based or protein-based data become available for *Drosophila*, our catalog of genes encoding short proteins remains incomplete.

In several vertebrate systems, ribosome profiling has been used to identify transcripts that appear to be translated and also the reverse, those that do not appear to be associated with the cytoplasmic translation machinery. Using mouse embryonic stem cells, Ingolia *et al.* (2011) found that the majority of transcripts previously identified as lncRNAs contained one or more short ORFs that were bound by elongating ribosomes. When this technique was expanded to detect small translated ORFs in UTRs, many were found, especially in 5′ UTRs (Ingolia *et al.* 2014). Using ribosome profiling data from zebrafish, combined with machine-learning analysis, Chew *et al.* (2013) also found that many previously identified lncRNAs appear to be protein-coding mRNAs. They identified an additional class, lncRNAs that have small ORFs that behave much like the uORFs found in 5′ UTRs, and postulate that ribosome association with this class of lncRNAs might be regulatory in nature.

Most FlyBase lncRNA annotations are based simply on evidence of transcription of the region. Currently, “lncRNA” is a very general categorization that includes short single-exon genes *vs.* genes that contain very long introns and extend hundreds of kilobases (see discussion of sex-specific genes above), genes that are almost entirely antisense and others that are antisense only in part, genes that appear to be the precursors of miRNAs, snoRNAs, or piRNAs, and genes that are tightly stage-specific or tissue-specific *vs.* those that are widely expressed. Our annotations of non-coding genes will evolve as knowledge of this relatively new field develops.

## Supplementary Material

Supporting Information
