# Supplementary material for: Gene Model Annotations for Drosophila melanogaster: Impact of High-Throughput Data
Source: G3 (Bethesda). 2015 Jun 24;5(8):1721–36. doi: 10.1534/g3.115.018929 (PMC4528329; doi:10.1534/g3.115.018929)
Supplement: Supporting Information [file supp_5_8_1721__index.html]

Gene Model Annotations for Drosophila melanogaster: Impact of High-Throughput Data — Supporting Information 

# Gene Model Annotations for *Drosophila melanogaster*: Impact of High-Throughput Data

## Supporting Information for Matthews *et al.*, 2015

**Files in this Data Supplement:**

- Supporting Information - Tables S1-S6 and descriptions of Files S1-S8 (PDF, 207 KB)
- Table S1 - FlyBase gene model and transcript comments. (PDF, 117 KB)
- Table S2 - FlyBase annotation IDs and the changes that occur to them as a result of annotation updates. (PDF, 112 KB)
- Table S3 - Improved UTR annotations in FlyBase annotation set R6.03. (PDF, 111 KB)
- Table S4 - Overlap of incorporated RNA-Seq exon junctions with annotated CDS, UTR and non-coding RNA. (PDF, 112 KB)
- Table S5 - Incorporation of modENCODE embryonic TSS regions into gene annotations. (PDF, 111 KB)
- Table S6 - Improvement of 3'UTR annotations. (PDF, 111 KB)
- File S1 - Gene model annotation correspondence between FlyBase R5.24 and R6.03. (.zip, 555 KB)
- File S2 - Improved UTR annotation of R5.24 protein coding transcripts in R6.03. (.zip, 2 MB)
- File S3 - Incorporation of RNA-Seq exon junction evidence into gene model annotations. (.zip, 1 MB)
- File S4 - Overlap of modENCODE TSS regions to R5.24 and R6.03 transcripts. (.zip, 455 KB)
- File S5 - Comparison of R5.24 and R6.03 3'UTR annotations. (.zip, 2 MB)
- File S6 - Small polypeptides. (.zip, 18 KB)
- File S7 - Sex-specific transcripts. (.zip, 337 KB)
- File S8 - Genes with known disruptive mutations in the reference genome assembly. (.zip, 11 KB)
