# Supplementary material for: Gene Model Annotations for Drosophila melanogaster: Impact of High-Throughput Data
Source: G3 (Bethesda). 2015 Jun 24;5(8):1721–36. doi: 10.1534/g3.115.018929 (PMC4528329; doi:10.1534/g3.115.018929)
Supplement: Supporting Information [file supp_g3.115.018929_018929SI.pdf]

G3 · Genes | Genomes | Genetics

Gene Model Annotations for *Drosophila melanogaster*: Impact of High-throughput Data

Beverley B. Matthews*[1], Gilberto dos Santos*, Madeline A. Crosby*, David B. Emmert*, Susan E. St. Pierre*, L. Sian Gramates*, Pinglei Zhou*, Andrew J. Schroeder*, Kathleen Falls*, Susan M. Russo*, William M. Gelbart*, and the FlyBase Consortium*[,†,‡,§,2]

*Department of Molecular and Cellular Biology, Harvard University, Cambridge, MA 02138
†Department of Genetics, University of Cambridge, Cambridge CB2 3EH, UK
‡Department of Biology, Indiana University, Bloomington, IN 47405
§Department of Biology, University of New Mexico, Albuquerque, NM 87131

[1]Corresponding author: Harvard University, Biological Laboratories, 16 Divinity Avenue, Cambridge, MA 02138. E-mail: bmatthew@morgan.harvard.edu

[2]The current members of the FlyBase Consortium are William Gelbart*, Nicholas H. Brown[†,] Thomas Kaufman[‡], Maggie Werner-Washburne[§], Richard Cripps[§], Phill Baker[§], Kris Broll*, Madeline Crosby*, Gilberto dos Santos*, David Emmert*, L. Sian Gramates*, Kathleen Falls*, Beverley B. Matthews*, Susan Russo*, Andrew Schroeder*, Pinglei Zhou*, Mark Zytkovicz*, Boris Adryan[†], Helen Attrill[†], Marta Costa[†], Steven Marygold[†], Peter McQuilton[†], Gillian Millburn[†], Laura Ponting[†], Raymund Stefancsik[†], Josh Goodman[‡], Gary Grumbling[‡], Victor Strelets[‡] and Jim Thurmond[‡].

DOI: 10.1534/g3.115.018929

SUPPORTING INFORMATION

Table S1. FlyBase gene model and transcript comments
Table S2. FlyBase annotation IDs and the changes that occur to them as a result of annotation updates
Table S3. Improved UTR annotations in FlyBase annotation set R6.03
Table S4. Overlap of incorporated RNA-Seq exon junctions with annotated CDS, UTR and non-coding RNA
Table S5. Incorporation of modENCODE embryonic TSS regions into gene annotations
Table S6. Improvement of 3'UTR annotations

File S1. Gene model annotation correspondence between FlyBase R5.24 and R6.03
File S2. Improved UTR annotation of R5.24 protein coding transcripts in R6.03
File S3. Incorporation of RNA-Seq exon junction evidence into gene model annotations
File S4. Overlap of modENCODE TSS regions to R5.24 and R6.03 transcripts
File S5. Comparison of R5.24 and R6.03 3'UTR annotations.
File S6. Small polypeptides
File S7. Sex-specific transcripts
File S8. Genes with known disruptive mutations in the reference genome assembly

Table S1 FlyBase gene model and transcript comments.

A. Gene model-associated comments.

Number of genes	Gene model-associated comment
596	Annotated transcripts do not represent all possible combinations of alternative exons and/or alternative promoters.
934	Annotated transcripts do not represent all supported alternative splices within 5' UTR.
4581	Low-frequency RNA-Seq exon junction(s) not annotated.
2272	Supported by strand-specific RNA-Seq data.
2112	Probable lncRNA gene; may encode small polypeptide(s).
138	Possible non-coding RNA gene.
322	Antisense: overlaps [] on opposite strand.
144	Antisense (in part): overlaps [] on opposite strand.
30	Gene model includes transcripts encoding non-overlapping portions of the full CDS.
51	Pseudogene similar to []; proximate; partial; created by tandem duplication.
101	Pseudogene similar to []; transposed.
6 / 4	Pseudogene similar to []; retrotransposed. / (may be retrotransposed)
54	Mutation in sequenced strain: [*].
5 / 6	Polymorphic pseudogene: intact in some individuals or strains, disrupted by mutation in others. / (may be polymorphic pseudogene)
3	Stop-codon suppression (UGA as Sec) postulated (FBrfnnnnnnn).
326	Stop-codon suppression (Uxx) postulated; FBrfnnnnnnn.
25	Unconventional translation start (XYZ) postulated; FBrfnnnnnnn.
2	Bidirectional region of coding sequence postulated: a portion of the CDS of this gene overlaps a portion of the CDS of a gene on opposite strand.

B. Transcript-associated comments.

Number of transcripts	Transcript-associated comments
23,858	Transcript terminates at site supported by polyadenylated cDNA.
2879	Extended 3' UTR based on RNA-Seq and/or EST data.
1990	UTR(s) based on RNA-Seq data.
1385	Transcriptional initiation is supported by short-capped RNA data (FBrf0209722).
1133	Transcriptional initiation is supported by RAMPAGE TSS data (FBrf0220331).

482	Evidence supports alternative splice leading to premature stop codon and/or downstream start; may or may not produce functional polypeptide.
139	Based on cDNA(s) with retained intron; results in premature stop codon and/or downstream start; may or may not produce functional polypeptide.
4	Stop-codon suppression (UGA as Sec) postulated (FBrfnnnnnnn); reflected in aa sequence of predicted polypeptide.
357	Stop-codon suppression (Uxx) postulated (FBrfnnnnnnn); reflected in aa sequence of predicted polypeptide.
58	Unconventional translation start postulated (XYZ encoding Met); FBrfnnnnnnn.
108	Downstream translation start supported by comparative analysis across Drosophila species.
14	Downstream translation start supported by [FBrfnnnnnnn].

Table S2 FlyBase annotation IDs and the changes that occur to them as a result of annotation updates.

Entity	Gene Symbol (examples)	Annotation Symbol (examples)	FlyBase ID prefix	Effect of annotation updates on Annotation IDs and FlyBase FBIDs	Fate of replaced IDs
gene	cnn<br>CG1354	CG4832<br>CG1354	FBgn	The annotation symbol and FBID both change when genes are merged or split. The non-CG gene symbol (if there is one) is retained (for one of the genes in the case of a split).	Old IDs are retained as synonyms (for symbols) or as secondary FBIDs
transcript	cnn-RA<br>cnn-RB<br>CG1354-RA<br>CG1354-RB	CG4832-RA<br>CG4832-RB<br>CG1354-RA<br>CG1354-RB	FBtr	Transcript symbols and IDs change if an annotation update alters the CDS in any way. The letter suffix is incremented to the next available. UTRs can be modified without changing IDs. Distinct transcripts can have the same CDS but differ only in their UTRs.	Old IDs are retained as synonyms (for symbols) or as secondary FBIDs
polypeptide	cnn-PA<br>cnn-PB<br>CG1354-PA<br>CG1354-PB	CG4832-PA<br>CG4832-PB<br>CG1354-PA<br>CG1354-PB	FBpp	The protein symbol suffix parallels the transcript symbol suffix and changes at the same time. As a result, proteins of identical amino acid sequence encoded by different transcripts have different IDs.	Old IDs are retained as synonyms (for symbols) or as secondary FBIDs

Table S3 Improved UTR annotations in FlyBase annotation set R6.03. The availability of high-throughput data made possible the annotation of UTRs for transcripts with sparse or no cDNA/EST support. The set of mRNA annotations in R5.24 (20,553 transcripts) and R6.03 (28,216 transcripts) were each assessed for the proportion of annotated transcripts lacking UTR annotations, and for the average/median size of annotated UTRs.

mRNA annotation characteristics	R5.24	R6.03
Percent of transcripts lacking 5'UTR only	3.4	0.8
Percent of transcripts lacking 3'UTR only	1.8	0.4
Percent of transcripts lacking both 5'UTR and 3'UTR	7.5	1.3
Percent of transcripts lacking either 5'UTR or 3'UTR	12.7	2.4
Average 5'UTR length (nt)	246	295
Median 5'UTR length (nt)	144	180
Average 3'UTR length (nt)	407	562
Median 3'UTR length (nt)	216	288

Table S4 Overlap of incorporated RNA-Seq exon junctions with annotated CDS, UTR and non-coding RNA. RNA-Seq exon junctions incorporated into FlyBase gene model annotations (i.e., matching an annotated intron) were assessed for their overlap to CDS, UTRs and non-coding RNA (lncRNA or pseudogene transcripts). Overlap was calculated separately for junctions that were "previously incorporated" in R5.24 (and therefore incorporated independently of RNA-Seq evidence), and for junctions that were "newly incorporated", based primarily on RNA-Seq data, subsequent to R5.24 (and still incorporated in R6.03).

	Percent of exon junctions overlapping*:			
Class of incorporated exon junction:	5'UTR	CDS	3'UTR	ncRNA
Newly incorporated exon junctions (n = 9,033)	37.3	46.2	4.4	12.1
Previously incorporated exon junctions (n = 46,319)	11.6	87.2	0.7	0.5

* Exon junctions that bridge a UTR and CDS, or that overlap different elements in different transcripts, were excluded from this tabulation. Also excluded from this tabulation were 254 exon junctions previously incorporated in R5.24 but subsequently withdrawn by R6.03. Results here are for 9,033 of 9,264 newly incorporated exon junctions, and 46,319 of 48,297 junctions previously incorporated in R5.24 and still incorporated R6.03.

Table S5 Incorporation of modENCODE embryonic TSS regions into gene annotations.
The overlap of high-confidence (“validated”) modENCODE embryonic TSS regions to annotated transcripts in R5.24 and R6.03. The percent of modENCODE TSS regions with the indicated kind of overlap to R5.24 and R6.03 transcripts is shown. See File S4 for details.

	Percent of modENCODE embryonic TSS regions (n = 8,678):	
Overlap of TSS to annotated transcripts:	R5.24	R6.03
Edge match of TSS 90% point and transcript 5’ end	2.8	90.0
TSS spans annotated transcript 5’ end (no edge match)	64.3	3.0
TSS overlaps transcript, but not transcript 5’ end	30.0	6.9*
TSS overlaps no transcripts	2.9	0.1

* The 595 cases in which a TSS region overlapped a transcript, but not its 5’ end, are currently being re-assessed with additional, independent TSS datasets.

Table S6 Improvement of 3’UTR annotations.

mRNA feature description	R5.24	R6.03
Number of mRNA with 3'UTR	19233	29867
Number of unique 3'ends	12887	17778
% with polyA cDNA support	45.3	73.6
Number of unique 3'UTRs*	13148	19471
Average size (nt)	393	555
Median size (nt)	213	250
% > 4kb	0.1	1.1
% 1-4kb	8.6	13.8
% < 1kb	91.3	85.0

*Excludes 387 (R5.24) and 537 (R6.03) unique 3'UTRs of transcripts within di/polycistronic genes.

Files S1-S8

Available for download at www.g3journal.org/lookup/suppl/doi:10.1534/g3.115.018929/-/DC1

File S1. Gene model annotation correspondence between FlyBase R5.24 and R6.03.
For each gene model, the annotation ID, FlyBase gene ID, gene symbol and gene class are shown in columns C-F for R5.24 and in columns H-K for R6.03, respectively. Annotations in R5.24 and R6.03 that have some relationship are listed in the same row, and the relationship is described in columns A and B. Column A classifies annotations in R5.24 and R6.03 as being "common" to both sets, related by gene merge, split or reclassification, or as being unique to R5.24 or R6.03. Additional descriptions of the relationship are provided in column B. Gene models encoding mRNA, ncRNA and pseudogene are shown in the first tab ("mRNA-ncRNA-pseudogene"), genes representing "small RNA"-encoding genes on the second tab. Note that although gene models will retain the same annotation ID in the absence of major structural reorganization or reclassification, there can be extensive changes in the number of transcript isoforms and the extent of the UTR annotations. Also note that only the net change after 26 annotation updates is shown; major changes to gene models may have taken place in several steps, and/or involved additional gene models created some time between R5.24 and R6.03 (these intermediates steps are not shown).

File S2. Improved UTR annotation of R5.24 protein coding transcripts in R6.03.
Transcripts for 13,104 protein coding gene models common to both R5.24 and R6.03 are listed; this set excludes transcripts of 9 gene models for which R5.24-R6.03 equivalence exists, but annotation IDs have changed (see File S1 for list and explanation). In column A, transcripts are indicated to be "common" to both R5.24 and R6.03, or specific to R5.24 or R6.03. The transcript annotation ID and FlyBase transcript ID are shown in columns B and C. mRNA, 5'UTR and 3'UTR length for R5.24 and R6.03 are shown in columns D-F and H-J. Columns G and K classify the UTR status of each transcript in R5.24 and R6.03, as having "no UTRs", "no 3'UTR", "no 5'UTR" or "both UTRs". The change (nucleotides) in mRNA, 5'UTR and 3'UTR size from R5.24 to R6.03 is calculated in columns L-N.

File S3. Incorporation of RNA-Seq exon junction evidence into gene model annotations.
All 71,514 RNA-Seq exon junctions obtained as evidence by FlyBase are listed in Column A by their FlyBase ID (FBsf#). The dataset(s) of origin for each exon junction is/are shown in Column B: Daines *et al.*, 2011 (BCM), Graveley *et al.*, 2011 (modENCODE), and unpublished lower confidence modENCODE junctions (modENCODE_Extra). The incorporation of each junction into a gene model ("Y" for yes, "N" for no) from annotation sets R5.24 and R6.03 is shown in Columns C and D, respectively. The overlap of each junction with 5' UTR, CDS and 3' UTR elements in R6.03 gene models is shown in columns E to G, respectively ("Y" for yes, "N" for no). Note that some junctions join a UTR to a CDS right at the UTR/CDS boundary; in these cases, overlap to both the CDS and UTR is scored as "Y", with an explanatory comment in Column I. Also note that a given junction can overlap different elements in different transcripts (for example, it may be found in the CDS of one transcript, and in the 5' UTR of another transcript). As such, the number of distinct ways that a junction overlaps transcript elements (i.e., overlap patterns) is indicated in Column H, with explanatory comments in Column I.

File S4. Overlap of modENCODE TSS regions to R5.24 and R6.03 transcripts.
For each high-confidence ("validated") modENCODE embryonic TSS region, its overlap to annotated transcripts in R5.24 and R6.03 was determined. Four kinds of overlap were scored; edge match - TSS 90% point matches the transcript 5' end; edge overlap - TSS spans annotated transcript 5' end (but 90% point does not match the transcript 5' end); other overlap - TSS overlaps the transcript but does not span the transcript 5' end; no overlap (to any transcripts). A single TSS region could overlap different transcripts in different ways: in these cases, only one type of overlap was reported according to the following ranking: edge match > edge overlap > other overlap. FlyBase TSS IDs are listed in Column A, overlap type to R5.24 and R6.03 transcripts in Columns B and C, respectively. Overlapping R5.24 and R6.03 transcripts in Columns D and E, respectively.

File S5. Comparison of R5.24 and R6.03 3'UTR annotations.
The unique set of annotated 3'UTRs is listed for R5.24 and R6.03 (on separate spreadsheet tabs). Each annotated 3'UTR was uniquely defined by its location (Column A). The 3'UTR's size and the location of its 3'end are listed in Columns B and C. The number of transcripts containing the indicated 3'UTR, and IDs for these transcripts, are listed in Columns D and E. 3'UTR ends that are supported by a polyadenylated cDNA, as determined by the associated transcript model comments, are indicated in Column F. Columns G and H list the annotation and FlyBase gene IDs for the gene associated with each distinct 3'UTR, with Column I indicating if the gene contains di/polycistronic transcripts. Column J indicates if the gene model is present in both R5.24 and R6.03 (as judged by an unchanged gene annotation ID).

File S6. Small polypeptides.
Predicted polypeptides of 50 residues or less from the R6.03 annotation set are listed.

File S7. Sex-specific transcripts.
As described in the text, the FlyBase RNA-Seq Search tool was used to identify 129 genes with female-specific expression and 2,414 genes with male-specific expression within the R6.03 annotation set. These genes are listed in separate tabs within the spreadsheet, along with additional information on gonadal expression (ovary or testis) and early embryo expression (for female-specific genes only).

File S8. Genes with known disruptive mutations in the reference genome assembly.
R6.03 gene model annotations with disruptive mutations in the genome assembly are listed. These disruptive mutations are typically specific to sequences derived from the "iso-1" reference strain. Gene IDs, annotation IDs and gene symbols are listed in Columns A-C respectively. The type of disruptive mutation is described in Column D. Columns E and F described any similarity of the "disrupted" gene to other protein coding genes in the R6.03 annotation set. Additional comments, associated alleles and transposon insertions are listed in Columns G-I, respectively.
