# Supplementary material for: Gene Model Annotations for Drosophila melanogaster: Impact of High-Throughput Data
Source: G3 (Bethesda). 2015 Jun 24;5(8):1721–36. doi: 10.1534/g3.115.018929 (PMC4528329; doi:10.1534/g3.115.018929)
Supplement: Supporting Information [file supp_g3.115.018929_TableS1.pdf]

Table S1 FlyBase gene model and transcript comments.

A. Gene model-associated comments.

Number of genes	Gene model-associated comment
596	Annotated transcripts do not represent all possible combinations of alternative exons and/or alternative promoters.
934	Annotated transcripts do not represent all supported alternative splices within 5' UTR.
4581	Low-frequency RNA-Seq exon junction(s) not annotated.
2272	Supported by strand-specific RNA-Seq data.
2112	Probable lncRNA gene; may encode small polypeptide(s).
138	Possible non-coding RNA gene.
322	Antisense: overlaps [] on opposite strand.
144	Antisense (in part): overlaps [] on opposite strand.
30	Gene model includes transcripts encoding non-overlapping portions of the full CDS.
51	Pseudogene similar to []; proximate; partial; created by tandem duplication.
101	Pseudogene similar to []; transposed.
6 / 4	Pseudogene similar to []; retrotransposed. / (may be retrotransposed)
54	Mutation in sequenced strain: [*].
5 / 6	Polymorphic pseudogene: intact in some individuals or strains, disrupted by mutation in others. / (may be polymorphic pseudogene)
3	Stop-codon suppression (UGA as Sec) postulated (FBrfnnnnnnn).
326	Stop-codon suppression (Uxx) postulated; FBrfnnnnnnn.
25	Unconventional translation start (XYZ) postulated; FBrfnnnnnnn.
2	Bidrectional region of coding sequence postulated: a portion of the CDS of this gene overlaps a portion of the CDS of a gene on opposite strand.

B. Transcript-associated comments.

Number of transcripts	Transcript-associated comments
23,858	Transcript terminates at site supported by polyadenylated cDNA.
2879	Extended 3' UTR based on RNA-Seq and/or EST data.
1990	UTR(s) based on RNA-Seq data.
1385	Transcriptional initiation is supported by short-capped RNA data (FBrf0209722).
1133	Transcriptional initiation is supported by RAMPAGE TSS data (FBrf0220331).

482	Evidence supports alternative splice leading to premature stop codon and/or downstream start; may or may not produce functional polypeptide.
139	Based on cDNA(s) with retained intron; results in premature stop codon and/or downstream start; may or may not produce functional polypeptide.
4	Stop-codon suppression (UGA as Sec) postulated (FBrfnnnnnnn); reflected in aa sequence of predicted polypeptide.
357	Stop-codon suppression (Uxx) postulated (FBrfnnnnnnn); reflected in aa sequence of predicted polypeptide.
58	Unconventional translation start postulated (XYZ encoding Met); FBrfnnnnnnn.
108	Downstream translation start supported by comparative analysis across Drosophila species.
14	Downstream translation start supported by [FBrfnnnnnnn].
