# Supplementary material for: Gene Model Annotations for Drosophila melanogaster: Impact of High-Throughput Data
Source: G3 (Bethesda). 2015 Jun 24;5(8):1721–36. doi: 10.1534/g3.115.018929 (PMC4528329; doi:10.1534/g3.115.018929)
Supplement: Supporting Information [file supp_g3.115.018929_TableS2.pdf]

Table S2 FlyBase annotation IDs and the changes that occur to them as a result of annotation updates.

Entity	Gene Symbol (examples)	Annotation Symbol (examples)	FlyBase ID prefix	Effect of annotation updates on Annotation IDs and FlyBase FBIDs	Fate of replaced IDs
gene	cnn<br>CG1354	CG4832<br>CG1354	FBgn	The annotation symbol and FBID both change when genes are merged or split. The non-CG gene symbol (if there is one) is retained (for one of the genes in the case of a split).	Old IDs are retained as synonyms (for symbols) or as secondary FBIDs
transcript	cnn-RA<br>cnn-RB<br>CG1354-RA<br>CG1354-RB	CG4832-RA<br>CG4832-RB<br>CG1354-RA<br>CG1354-RB	FBtr	Transcript symbols and IDs change if an annotation update alters the CDS in any way. The letter suffix is incremented to the next available. UTRs can be modified without changing IDs. Distinct transcripts can have the same CDS but differ only in their UTRs.	Old IDs are retained as synonyms (for symbols) or as secondary FBIDs
polypeptide	cnn-PA<br>cnn-PB<br>CG1354-PA<br>CG1354-PB	CG4832-PA<br>CG4832-PB<br>CG1354-PA<br>CG1354-PB	FBpp	The protein symbol suffix parallels the transcript symbol suffix and changes at the same time. As a result, proteins of identical amino acid sequence encoded by different transcripts have different IDs.	Old IDs are retained as synonyms (for symbols) or as secondary FBIDs
