# Supplementary material for: Gene Model Annotations for Drosophila melanogaster: Impact of High-Throughput Data
Source: G3 (Bethesda). 2015 Jun 24;5(8):1721–36. doi: 10.1534/g3.115.018929 (PMC4528329; doi:10.1534/g3.115.018929)
Supplement: Supporting Information [file supp_g3.115.018929_TableS3.pdf]

Table S3 Improved UTR annotations in FlyBase annotation set R6.03. The availability of high-throughput data made possible the annotation of UTRs for transcripts with sparse or no cDNA/EST support. The set of mRNA annotations in R5.24 (20,553 transcripts) and R6.03 (28,216 transcripts) were each assessed for the proportion of annotated transcripts lacking UTR annotations, and for the average/median size of annotated UTRs.

mRNA annotation characteristics	R5.24	R6.03
Percent of transcripts lacking 5'UTR only	3.4	0.8
Percent of transcripts lacking 3'UTR only	1.8	0.4
Percent of transcripts lacking both 5'UTR and 3'UTR	7.5	1.3
Percent of transcripts lacking either 5'UTR or 3'UTR	12.7	2.4
Average 5'UTR length (nt)	246	295
Median 5'UTR length (nt)	144	180
Average 3'UTR length (nt)	407	562
Median 3'UTR length (nt)	216	288
