# Supplementary material for: Gene Model Annotations for Drosophila melanogaster: Impact of High-Throughput Data
Source: G3 (Bethesda). 2015 Jun 24;5(8):1721–36. doi: 10.1534/g3.115.018929 (PMC4528329; doi:10.1534/g3.115.018929)
Supplement: Supporting Information [file supp_g3.115.018929_TableS4.pdf]

Table S4 Overlap of incorporated RNA-Seq exon junctions with annotated CDS, UTR and non-coding RNA. RNA-Seq exon junctions incorporated into FlyBase gene model annotations (i.e., matching an annotated intron) were assessed for their overlap to CDS, UTRs and non-coding RNA (lncRNA or pseudogene transcripts). Overlap was calculated separately for junctions that were "previously incorporated" in R5.24 (and therefore incorporated independently of RNA-Seq evidence), and for junctions that were "newly incorporated", based primarily on RNA-Seq data, subsequent to R5.24 (and still incorporated in R6.03).

	Percent of exon junctions overlapping*:			
Class of incorporated exon junction:	5'UTR	CDS	3'UTR	ncRNA
Newly incorporated exon junctions (n = 9,033)	37.3	46.2	4.4	12.1
Previously incorporated exon junctions (n = 46,319)	11.6	87.2	0.7	0.5

* Exon junctions that bridge a UTR and CDS, or that overlap different elements in different transcripts, were excluded from this tabulation. Also excluded from this tabulation were 254 exon junctions previously incorporated in R5.24 but subsequently withdrawn by R6.03. Results here are for 9,033 of 9,264 newly incorporated exon junctions, and 46,319 of 48,297 junctions previously incorporated in R5.24 and still incorporated R6.03.
