# Supplementary material for: Gene Model Annotations for Drosophila melanogaster: Impact of High-Throughput Data
Source: G3 (Bethesda). 2015 Jun 24;5(8):1721–36. doi: 10.1534/g3.115.018929 (PMC4528329; doi:10.1534/g3.115.018929)
Supplement: Supporting Information [file supp_g3.115.018929_TableS5.pdf]

Table S5 Incorporation of modENCODE embryonic TSS regions into gene annotations.
The overlap of high-confidence ("validated") modENCODE embryonic TSS regions to annotated transcripts in R5.24 and R6.03. The percent of modENCODE TSS regions with the indicated kind of overlap to R5.24 and R6.03 transcripts is shown. See File S4 for details.

	Percent of modENCODE embryonic TSS regions (n = 8,678):	
Overlap of TSS to annotated transcripts:	R5.24	R6.03
Edge match of TSS 90% point and transcript 5' end	2.8	90.0
TSS spans annotated transcript 5' end (no edge match)	64.3	3.0
TSS overlaps transcript, but not transcript 5' end	30.0	6.9*
TSS overlaps no transcripts	2.9	0.1

* The 595 cases in which a TSS region overlapped a transcript, but not its 5' end, are currently being re-assessed with additional, independent TSS datasets.
