# Supplementary material for: Gene Model Annotations for Drosophila melanogaster: Impact of High-Throughput Data
Source: G3 (Bethesda). 2015 Jun 24;5(8):1721–36. doi: 10.1534/g3.115.018929 (PMC4528329; doi:10.1534/g3.115.018929)
Supplement: Supporting Information [file supp_g3.115.018929_TableS6.pdf]

Table S6 Improvement of 3’UTR annotations.

mRNA feature description	R5.24	R6.03
Number of mRNA with 3'UTR	19233	29867
Number of unique 3'ends	12887	17778
% with polyA cDNA support	45.3	73.6
Number of unique 3'UTRs*	13148	19471
Average size (nt)	393	555
Median size (nt)	213	250
% > 4kb	0.1	1.1
% 1-4kb	8.6	13.8
% < 1kb	91.3	85.0

*Excludes 387 (R5.24) and 537 (R6.03) unique 3'UTRs of transcripts within di/polycistronic genes.
